# Transcriptome and 2-DE proteome analyses reveal defense-associated development in the leaf galls induced by psyllids on *Machilus japonica* var. *kusanoi*

**DOI:** 10.1186/s40529-025-00470-2

**Published:** 2025-07-14

**Authors:** Tin-Han Shih, Jian-Liang Chen, Meng-Yuan Huang

**Affiliations:** 1 Department of Life Sciences, National Chung Hsing University, Taichung, Taiwan; 2 Innovation and Development Center of Sustainable Agriculture, National Chung Hsing University, 145 Xingda Rd., South Dist, Taichung City, 402202 Taiwan

**Keywords:** Galls, Plant defense, *Machilus japonica* var. *kusanoi*, Psyllids, ROS, Transcriptome

## Abstract

**Background:**

Galls are abnormal plant tissues that result from the interaction between the defense responses of plants and the activities of galling herbivores. During infection, secondary metabolites are synthesized to mitigate the oxidative stress whereas growth and cellular processes in infected tissues are reprogrammed. Although the regulatory networks of growth-related phytohormones are recognized as the main component in gall formation, other factors like oxidative stress might also being critical in gall development. In this study, we focused on the spherical psyllid galls found in the leaves of *Machilus japonica* var. *kusanoi* to analyze the differences in transcript, protein content, and several metabolites between galls and leaves.

**Results:**

Early gall tissues were enriched in cell cycle regulation and organization genes, suggesting processes, such as mitosis, meiosis, and microtubule-based movement. Proteins such as CAM7, LSH6, and eIF2β, associated with seed development, were highly expressed in early gall tissues. We demonstrated a significant role for ROS-related stress responses in early gall development. Higher amount of polyphenols, which are well-known antioxidants, was found in gall tissue as well.

**Conclusions:**

This study provides valuable insights into the mechanisms underlying gall development and enhances our understanding of this complex process. Future research should explore the impact of ROS modulation on gall tissue development and assess phytohormone content at various psyllid larval and gall stages to elucidate the regulatory network involved in gall morphogenesis.

**Supplementary Information:**

The online version contains supplementary material available at 10.1186/s40529-025-00470-2.

## Introduction

Galls are unique plant tissues formed in response to various parasites and exhibit a wide range of morphologies. The formation of galls, initiated by various galling organisms like insects, induces complex physiological responses in host plants. This interaction triggers a cascade of events, including genetic reprogramming, ectopic cell proliferation and expansion, influencing the host’s physiology and metabolism (Raman 2021). It was considered that gall inducers manipulate host plant tissues via the changes of phytohormones. Accumulation of auxin and cytokinin at insect-infected sites and within developing gall tissues has been observed across various plant species (Mapes and Davies 2001; Chen et al. 2009; Tooker and De Moraes 2011). Transcriptomic profiling of gall tissues revealed increased expression of genes associated with auxin and cytokinin biosynthesis and signaling pathways, indicating their role in regulating gall growth (Bailey et al. 2015; Shih et al. 2018; Schultz et al. 2019). Insect larvae have been found to synthesize and secrete these phytohormones, influencing tissue development and allowing them to manipulate plant networks (Yamaguchi et al. 2012; Tanaka et al. 2014; Tooker and Helms 2014; Hirano et al. 2020).

In addition to insect manipulation, plant stress and defense responses play significant roles in gall development. Innate immune responses, including pattern-triggered immunity (PTI), influence cell cycle progression, expansion, and proliferation, resembling gall-forming features (Harris and Pitzschke 2020). Recent studies have suggested the critical roles of reactive oxygen species (ROS) in gall development. ROS, particularly superoxide ion and hydrogen peroxide, are implicated in eliciting hypersensitive responses (HR) in plants, which involve oxidative bursts around the oviposition and feeding sites of galling insects (Oliveira and Isaias 2010; Isaias et al. 2015; Oates et al. 2020; Guedes et al. 2022). The compartments of insect galls exhibit distinctive ROS levels, with inner tissue compartments (IC) primarily associated with nutritive functions and outer tissue compartments (OC) linked to defense and maintenance of gall structure (Guedes et al. 2022). Transcriptomic analysis of the oviposition spot of Eucalyptus revealed the enrichment of genes associated with the responses to oxidative stress and stimulus of phytohormones such as auxin, jasmonic acid (JA), and salicylic acid (SA), suggesting a synergic activity of these molecules in the gall initiation stage (Oates et al. 2020). Although these researches have referred the role of ROS in gall tissues, extensive molecular analysis still needed to explore the profound contribution of oxidative stress to gall development.

Although respond to stress in plant is considered to be a universal reaction after infection, there are still have different regulatory pattern underneath among different galler-host plant interaction. In mite-induced galls on Chinese sumac (*Rhus javanica*), the genes of transcription factors that regulate meristem development have been showed a strongly increase in expression (Hirano et al. 2020). Other study in phylloxera-grapevine galling system found that the expression of reproductive genes has been reprogrammed during gall development (Schultz et al. 2019). Transcriptomic analysis in leaves of *Metrosideros polymorpha*, on the other hand, have identified a significant enrichment of auxin responding-genes after galling by psyllid (Bailey et al. 2015).

Psyllids are a family known for inducing galls, which can vary from simple leaf curls to complex structures (Hodkinson 2009; Sharma and Raman 2022). Eggs are deposited in cracks on the bark of terminal twigs prior to leaf flushing. Once the leaves emerge, the nymphs migrate to the newly flushed foliage. The feeding strategies of gall-inducing psyllids induce metabolic changes in the plant, allowing continued development within galls. Feeding injects saliva and enzymes into plant tissues, causing symptoms resembling premature senescence and cell degeneration (Crawford and Wilkens 1996). The literature has demonstrated gene expression alterations in psyllid-infected leaves; however, the examination of molecular regulation in psyllid gall tissues remains insufficient. In present study, therefore, we attempt to characterize the gene regulatory mechanisms of the globular galls induced by psyllid (*Neotrioza shuiliensis*) on the leaves of *Machilus japonica* var. *kusanoi* (Lauraceae). We analyzed the transcriptome to reveal the enrichment of biological functions in galls at early and late (mature) developmental stages. We also performed two-dimensional gel electrophoresis (2-DE) to identify proteins that may regulate early gall formation.

In our study, early gall tissues were enriched with differentially expressed genes (DEGs) involved in the cell cycle, signaling, and microtubule-based processes. In contrast, mature galls showed enrichment in DEGs associated with nutrient catabolic processes. Both stages of gall development exhibited DEG enrichment related to hydrogen peroxide metabolism. Protein analysis identified several proteins responsive to reactive oxygen species (ROS) and plant programmed cell death (PCD) that were increased in developing galls. Proteins directly responding to auxin may also contribute to ROS detoxification. These findings suggest that the preferential response to stress signals, such as ROS, is the primary factor initiating and mediating psyllid gall development.

## Materials and methods

### Plant materials

**Fig. 1:**
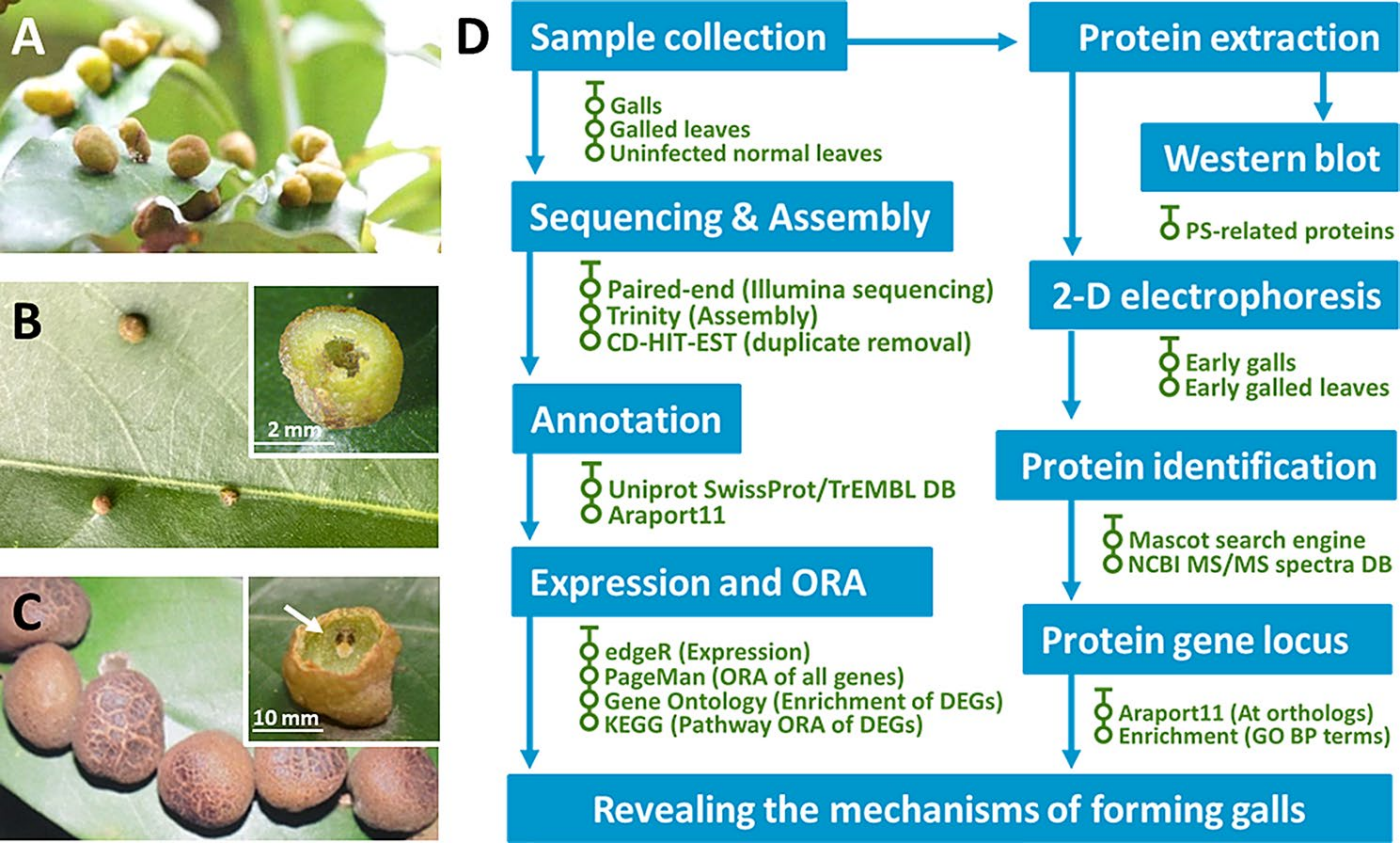
Sample photos and research scheme. (**A**). Globular galls induced by *Neotrioza shuiliensis* on the leaves of *Machilus japonica var. kusanoi.* (**B**). Galls in the early developmental stages. Larva was at 2nd instar). (**C**) Galls in the late/mature developmental stages. Larva was at the 5th instar and indicated by the arrow. (**D**) The scheme of bioinformatic experiment proceeded in the present study

*Machilus japonica* var. *kusanoi* leaf galls of psyllid *Neotrioza shuiliensis* were harvested from 3 different trees at Erziping, Yangmingshan National Park in Taiwan, in 2019–2021. In May, galls inhabited with 2nd or 3rd instar larvae were collected as early-stage gall tissues (E-G) under remarkable tissue growth (Fig. 1A-B). In November, galls inhabited with 5th instar larvae were collected as late-stage gall tissues (L-G), which are mature and show no apparent gall size enlargement (Fig. 1C). The infected leaves with E-G and L-G were collected and referred to as early and late-galled leaves (E-GL and L-GL). For reference, the uninfected normal leaves on the adjacent branches were also collected as early and late normal leaves (E-L and L-L). Only healthy galls were collected to prevent the regulatory interference induced by parasites or premature gall abortion. All samples were briefly cleaned and frozen in liquid nitrogen on the field before being stored at -80℃.

### RNA extraction and cDNA library Preparation

For RNA sequencing and transcriptome analysis, total RNA from galls and leaves was prepared by the cetyltrimethylammonium bromide (CTAB)-based method (MacRae 2007). A hundred mg of tissue powder was subjected to CTAB extraction buffer. Purified RNA was stored at -80℃ for the following experiments.

Poly-T oligo-attached beads were used to purify mRNA for cDNA synthesis. Random primer was used to synthesize first-strand cDNA, followed by generating double-strand cDNA with dTTP replacement by dUTP. After quality checked by Bio-analyzer (Agilent 2100, CA, USA), three cDNA libraries each for E-G, L-G, E-GL, L-GL, E-L, and L-L were prepared for 150 bp paired-end RNA-Seq transcriptome analysis.

### Sequencing and de Novo assembly

Sequencing of cDNA was performed using the Illumina HiSeq™ 4000 platform (SRA submission ID: SUB14169900). Trimmomatic was used for trimming adaptors and removing low-quality reads (QV < 20). *De novo* assembly was completed through Trinity (v2.3.2, (Haas et al. 2013) with the parameter of minimum contig length ≧ 300 bps. After assembly, CD-HIT-EST (Fu et al. 2012) was carried out to remove redundant transcripts and collect more specific unigenes (sequence identity threshold ≧ 95%). cDNA library construction, sequencing, and *de novo* assembly were performed by commercial service (Genomics, New Taipei City, Taiwan).

### Gene quantification, annotation, and DEGs identification

After the alignment of reads by bowtie2 Field (Langmead and Salzberg 2012), quantification of the read count was calculated by RSEM (v1.2.31, (Li and Dewey 2011). Transcript levels were further normalized as fragments per kilobase of the exon model per million mapped reads (FPKM) and per kb per million reads (RPKM). All contigs were blasted against the Uniprot SwissProt/TrEMBL database through Trinotate (v3.0.2, https://trinotate.github.io/). For functional enrichment analysis, the accession IDs of *M. kusanoi* orthologous genes in Arabidopsis were obtained through blasting against the Arabidopsis database (Araport 11). Differential expressed genes (DEGs) in each comparison were calculated by edgeR v3.5 (McCarthy et al. 2012) and were filtered by a false discovery rate (FDR) of 0.05 and a log2 fold change (Log2FC) threshold of 2.

### Functional enrichment analysis

To specifically examine the function of gene groups, *M. kusanoi* genes were subjected to the platforms that support Arabidopsis gene loci. Before that, TAIR-annotated genes with the highest bit score were kept when multiple genes were mapped onto duplicate orthologs. The present study used PageMan (integrated into MapMan ver.3.5.1 Field, (Usadel et al. 2006) to perform the overrepresentation analysis (ORA) of gene functions in gall and leaf tissues. PageMan ORA was achieved by Benjamini and Hochberg FDR methods (Benjamini and Hochberg 2018) with a cutoff value 2. This study also used the online platform WEB-based Gene SeT AnaLysis Toolkit WebGestalt, (Liao et al. 2019) for the gene ontology (GO) and KEGG pathway enrichment analysis of DEGs in each comparison. Significant GO and KEGG pathway enrichment of DEGs were set as FDR < 0.05 (B-H method).

### Two-dimensional gel electrophoresis (2-DE)

To identify the proteins induced during gall development, the proteome of gall tissues in the early stage was analyzed using a 2-D electrophoresis (2-DE)-based approach. Eight protein spots from the E-G gel were screened for protein identification because of either elevated protein amounts or newly synthesized proteins compared to the 2-DE of E-GL. Three galls on a same leaf were homogenized together as an E-G sample for 2-DE. The corresponding host leaf was prepared as sample E-GL. Total proteins of gall tissue and leaf were extracted using a plant protein extraction kit (PE0230, Sigma-Aldrich). 500 µg of total proteins were separated by 13 cm Immobiline DryStrip pH 3–10 non-linear on the isoelectric focusing system (Ettan IPGphor 3 IEF system, GE Healthcare Life Science) in the first dimension (total 7,400 Vhr). Following equilibration by incubating with 65 mM dithiothreitol and 135 mM iodoacetamide), the IPG gel strips were transferred onto vertical gels (12% SDS-PAGE, Hoefer SE600, GE Healthcare Life Science) for the second-dimension separation and visualized with Start Blue staining. The selected gel spots were pulled together and digested by trypsin (Promega). Peptides were then dried and reconstituted with 0.5% formic acid for the following procedures.

### Liquid chromatograph/tandem mass spectrometer (LC-MS/MS) analysis

Tryptic-digested peptides were diluted in HPLC buffer A (0.1% formic acid) and loaded onto a reverse phase column (Zorbax 300SB C18, 0.3 × 5 mm, Agilent). The desalted peptides were then separated on a homemade column (HydroRP 2.5 μm, 75 μm I.D. × 20 cm with a 15 μm tip) using a multi-step gradient of HPLC buffer B (99.9% acetonitrile/0.1% formic acid) for 70 min with a flow rate of 0.3 µl min^− 1^. The LC apparatus was coupled with a 2D linear ion trap mass spectrometer (Orbitrap Elite ETD; Thermo Fisher) operated using Xcalibur 2.2 software (Thermo Fisher). The full scan MS was performed in the Orbitrap over 400 to 2,000 Da and a resolution of 120,000 at m/z 400. The 20 data-dependent MS/MS scan events were followed by one MS scan for the 20 most abundant precursor ions in the preview MS scan. The m/z values selected for MS/MS were dynamically excluded for 40 s with a relative mass window of 15 ppm. The electrospray voltage was set to 2.0 kV, and the capillary temperature was set to 200 °C. MS and MS/MS automatic gain control was set to 1,000 ms (full scan) and 200 ms (MS/MS), or 3 × 10^6^ ions (full scan) and 3,000 ions (MS/MS) for maximum accumulated time or ions, respectively.

### Protein identification

The MS/MS spectra were searched against the NCBI database using the Mascot search engine (Matrix Science, London, UK; version 2.5). For peptide identification, 10 ppm mass tolerance was permitted for intact peptide masses and 0.5 Da for CID fragment ions with allowance for two missed cleavages made from the trypsin digestion. Peptide spectrum match (PSM) were then filtered based on high confidence and Mascot search engine rank 1 of peptide identification to ensure an overall false discovery rate below 0.01. Proteins with single peptide hits were removed.

### ROS detection of gall and leaves

To detect ROS in gall tissues, 3,3’-diaminobenzidine (DAB, Sigma) was introduced to early-stage galls to illustrate the in-situ distribution of hydrogen peroxide. Freshly detached galls (*n* = 5) and corresponding galled leaves (*n* = 3) were immersed in a DAB staining solution (1 mg ml^− 1^, pH 3.8) for 8 h at room temperature and were subsequently incubated in 100% ethanol until chlorophyll and unprecipitated stains were cleared. Precipitations in galls and galled leaves were then observed under a microscope.

### Photopigments and polyphenols content

#### Chlorophyll content

To test the content of chlorophyll a (Chl a) and chlorophyll b (Chl b) in gall and leaf tissues, 100 mg of samples. were ground in liquid nitrogen and were immersed in 1 mL acetone (80% v/v) at 4℃. Each gall sample contains tissues homogenized form 1–2 galls whereas a leaf sample has the tissue form single leaflet. After centrifuge (4,000 rpm, 5 min), supernatant was collected for the measurement of absorption spectrum. Absorption of the extracts was examined at 663.6 nm and 646.6 nm for detecting Chl a and Chl b, respectively. The content of Chl a and Chl b were determined in accordance with Yang et al. (Yang et al. 1998) as follow:

$$\eqalign{ & \>Chl\>a = 12.25 \cdot \>A663.6\> - \>2.55 \cdot \>A646.6\> \cr & \times \>\>supernatant\>V\>\left( {mL} \right)/sample\>weight\>\left( g \right) \cr} $$

$$\eqalign{ & \>Chl\>b = 20.31 \cdot \>A646.6\> - \>4.91 \cdot \>A663.6 \cr & \> \times \>\>supernatant\>V\>\left( {mL} \right)/sample\>weight\>\left( g \right) \cr} $$

*A*_663.6_ and *A*_646.6_ are the absorbance of sample extracts measured at 663.6 nm and 646.6 nm, respectively.

### Anthocyanin content

To determine the content of anthocyanin, 100 mg of sample tissues were ground in liquid nitrogen and then immersed in 2 ml methanol (1% HCl). Each gall sample contains tissues homogenized form 1–2 galls whereas a leaf sample has the tissue form single leaflet. Supernatant was collected after centrifuge (8000*g*, 10 min) and 4℃. The content of anthocyanin was calculated according to Mancinelli et al. (1975) as follow:

$$\eqalign{ & \>Anthocyanin\> = \>(A530\> - \>0.33 \cdot \>A657/31.6)\> \cr & \times \>\>supernatant\>V\>\left( {ml} \right)/sample\>weight\>\left( g \right) \cr} $$

*A*_530_ – 0.33∙*A*_657_ are the absorbances at 530 nm and 657 nm, respectively.

All absorbances were measured by Hitachi U2800 spectrophotometer (Hitachi, Japan).

### Flavonoid content

The flavonoid content was determined following the method described by Lesjak et al. (2013). A range of 4 mg/mL to 11.5 mg/mL of dried sample was ground and subjected to flavonoid extraction using 90% methanol. After centrifugation, 30 µl of the supernatant was diluted with 90 µl of methanol, followed by the addition of 6 µl of 10% aluminum chloride (AlCl_3_, Sigma-Aldrich) and 6 µl of 1 M sodium acetate (CH_3_COONa, Sigma-Aldrich). The absorbance was measured at 415 nm after adding 170 µl of distilled water and allowing the mixture to stand for 30 min. The total flavonoid content was expressed as milligrams of quercetin equivalents (QE) per gram of dried sample weight (DW).

### Polyphenol content

The polyphenol contents in the collected samples were analyzed using the method established by Singleton and Rossi (1965). Briefly, 90% methanol with 0.3% (v/v) HCl was added to 10 mg of powdered dried sample. One gall or 1 sliced leaflet represents 1 gall or leaf sample, respectively. Following centrifugation, 100 µL of the supernatant was mixed with 2 mL of a 2% Na_2_CO_3_ solution, and the absorbance was measured at 750 nm using a spectrophotometer. Gallic acid (Sigma) was used to construct the calibration curve. The concentration of polyphenols was determined and expressed as gallic acid equivalents (GAE).

## Results

### Statistic of sequencing, de novo assembly, and gene annotation

Six groups of samples, including the early and late developmental stage of gall tissues (E-G and L-G), their host leaves (E-GL and L-GL), and the corresponding uninfected normal leaves (E-L and L-L) in *M. kusanoi*, were collected to examine changes in the transcriptome profiles of leaf galls during development. The experimental scheme is illustrated in Fig. 1D. Transcripts from six groups of samples were paired-end sequenced, and RNA-seq data were *de novo* assembled by combining Trinity and CD-HIT-EST to recover more specific genes. The assembly of approximately 964 million paired-end reads generated 550,506 raw contigs with a minimum length of 300 base pairs (Table S1; Fig. S1). A total of 478,892 contigs were derived from raw assembled transcripts but clustered with 95% similarity (Table S2). All 478,892 Trinity contigs were searched against the Arabidopsis reference genome using BLASTx, and 438,906 contigs were annotated (Datasheet S1). Gene expression analysis of the RNA-seq expression profiles in galls and leaves was performed using edgeR (v3.5) with the trimmed mean of M-values (TMM) method.

### Correlation of galls and leaves samples

**Fig. 2:**
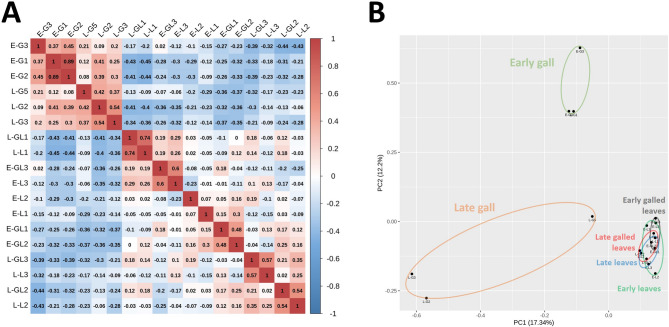
Correlation of sequenced galls and their infected and uninfected leaves. (**A**) Pearson correlation coefficient diagram of sample libraries. (**B**) Principal component analysis (PCA) of all sequenced samples. E-G, early-stage galls; L-G, late/mature stage galls; E-GL or L-GL, galled leaves corresponding to early or late gall stages, respectively; E-L or L-L, normal non-galled leaves corresponding to early or late gall stage, respectively

The correlation score in the matrix indicated that galls in the early stage (E-Gs) or late stage (L-Gs) were clustered (Fig. 2A). However, the corresponding leaf samples (early or late stages) showed no distinguishable clustering regardless of whether the leaves were infected. As observed by principal component analysis (PCA), gene expression patterns in E-Gs and L-Gs were distinct from those observed in host and uninfected leaves (Fig. 2B). PCA also grouped E-Gs and L-Gs into different clusters, whereas all the leaf groups overlapped.

### Overrepresentation analysis of all genes

**Fig. 3:**
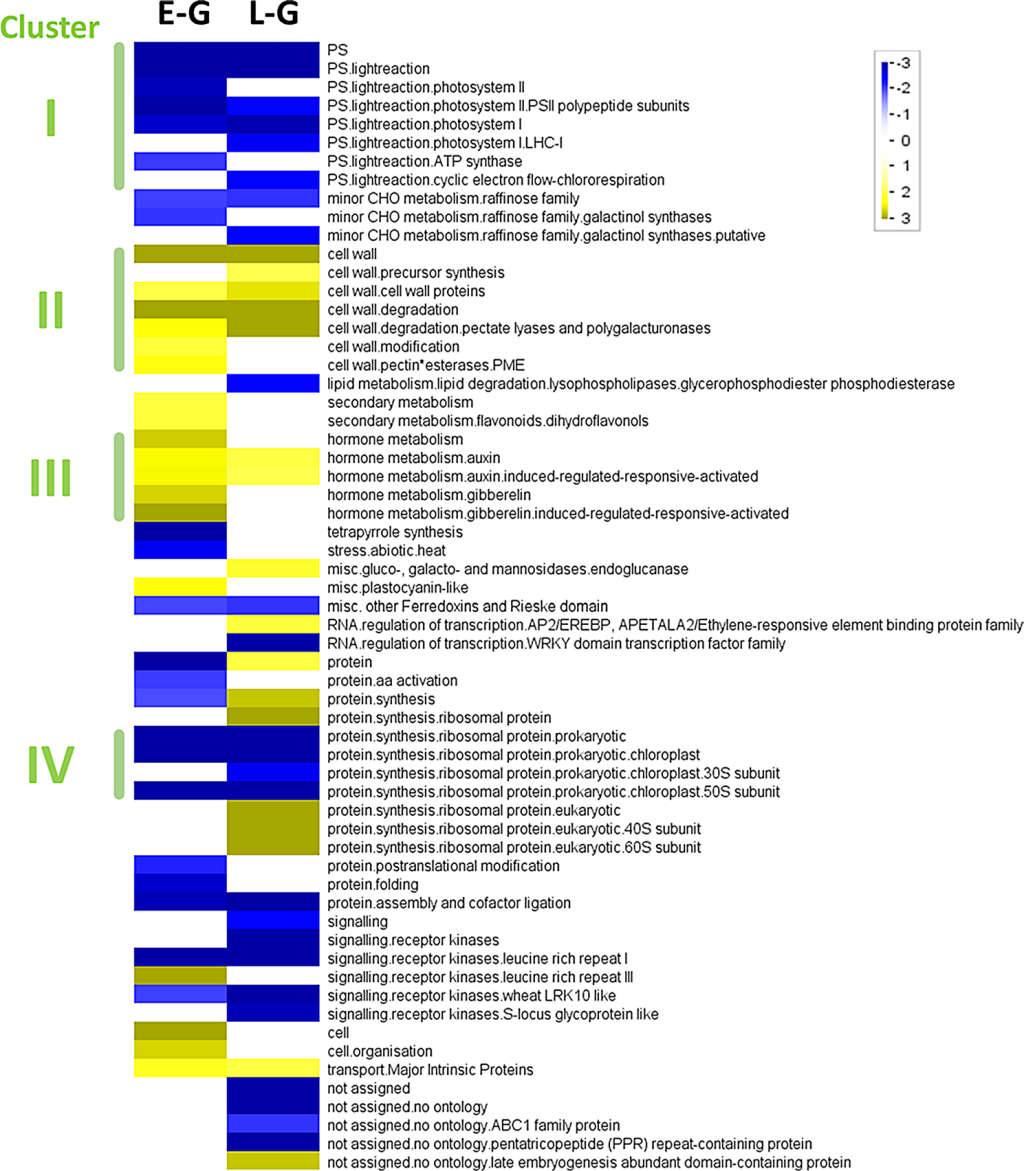
Overrepresentation analysis of functional enrichments in gall tissues at early and late developmental stages. All genes annotated to Arabidopsis orthologs were subjected to PageMan for ORA. Comparison of gene expression between gall tissues and galled leaves were input as the expression profiles. Lane E-G, comparison of E-G over E-GL. Lane L-G, comparison of L-G over L-GL. Blue bins, down-regulated in gall tissues. Yellow bins, up-regulated in gall tissue. E-G, early-stage galls; L-G, late/mature stage galls

When compared with the host leaves, early-stage galls (E-G) and late-stage galls (L-G) were both enriched with genes involved in cell wall synthesis/degradation and hormonal metabolism (Fig. 3, clusters II and III). In contrast, genes involved in photosynthesis or chloroplast-associated protein synthesis were significantly downregulated at both gall stages (Fig. 3, clusters I and IV). The bins showed that genes involved in gibberellin metabolism and cell organization were enriched in early gall tissues. Genes involved in protein synthesis and modification were downregulated in the E-G.

**Fig. 4:**
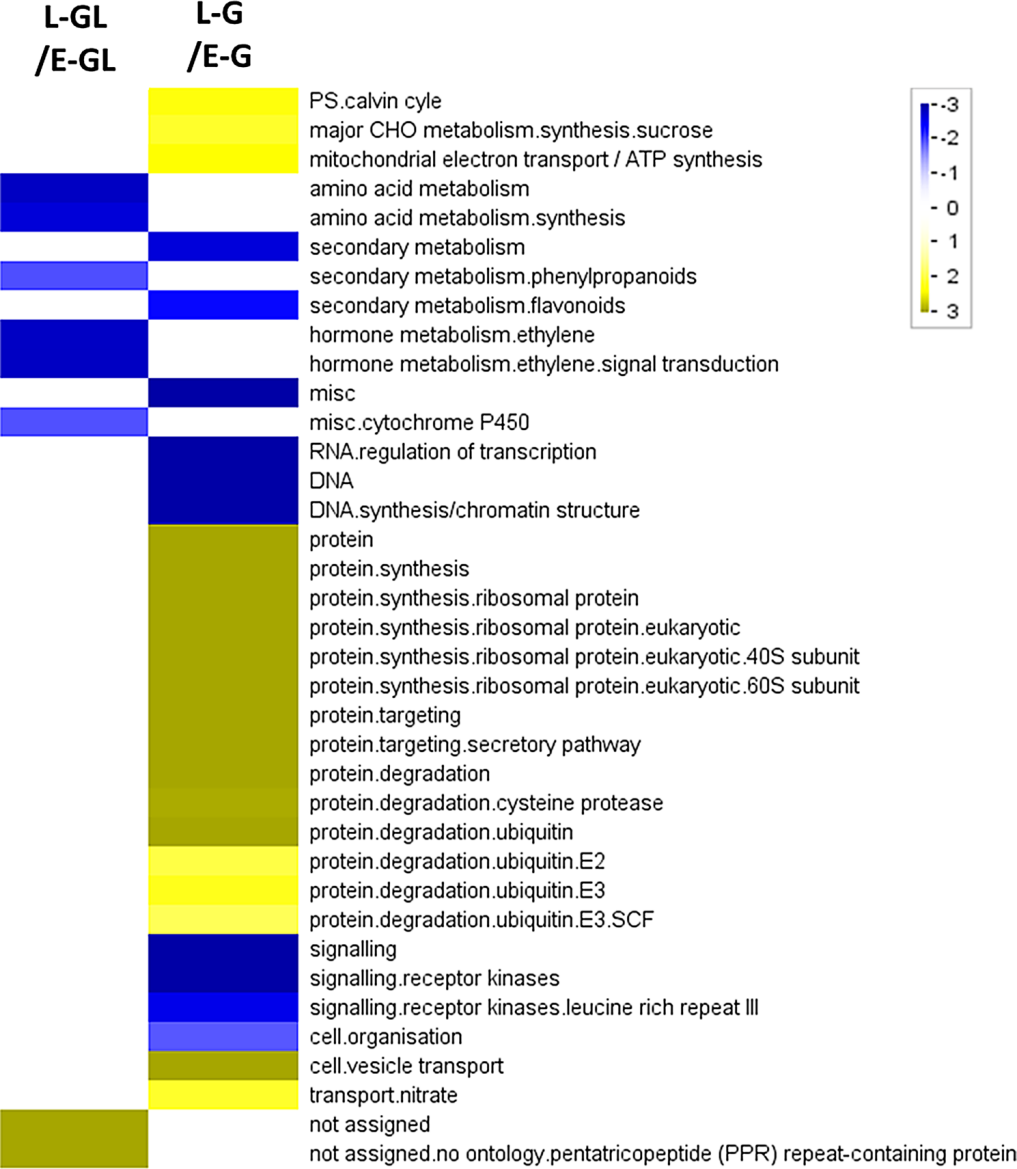
Overrepresentation analysis of functional enrichments in different stages of gall tissues and corresponding galled leaves. In lane L-GL/E-GL, blue bin indicates the functional enrichment in E-GL whereas yellow bin indicates the functional enrichment in L-GL. In lane L-G/E-G, blue bin indicates the functional enrichment in E-G whereas yellow bin indicates the functional enrichment in L-G. E-G, early stage galls; L-G, late/mature stage galls; E-GL or L-GL, galled leaves corresponding to early or late gall stages, respectively

The data were also subjected to functional enrichment analysis between the early and late stages of galls. Early galls were enriched in genes regulating DNA synthesis, RNA transcription, receptor-like kinase signaling, and cell organization (Fig. 4, blue bins in lane L-G/E-G). When developing into the late/mature stage, the galls were enriched in genes involved in protein metabolism (both synthesis and degradation) and vesicle transportation (Fig. 4, yellow bins in lane L-G/E-G). Notably, late-stage galls were enriched in genes associated with the Calvin cycle, sucrose synthesis, and mitochondrial ATP synthesis compared to early-stage galls.

### Screening and retrieving AGI-IDs of differentially expressed genes (DEGs)

**Fig. 5:**
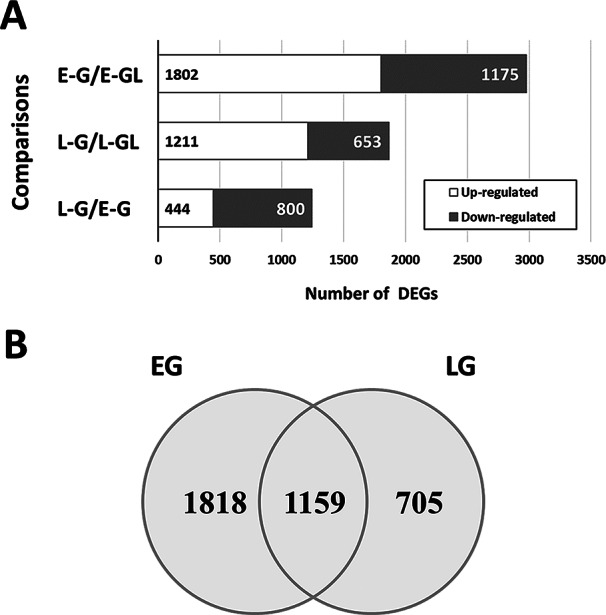
Numbers of differential expressed genes (DEGs). (**A**) Numbers of up- and down-regulated DEGs in different stages of galls. (**B**) Venn diagram showing the distribution of shared and unique DEGs found in different gall stages. E-G, early-stage galls; L-G, late/mature stage galls; E-GL or L-GL, galled leaves corresponding to early or late gall stages, respectively. Details of DEGs were listed in Datasheet S2

The Arabidopsis gene ID (AGI) of the DEGs in the galls and leaves was retrieved after removing AGI replicates among the genes (Datasheet S2). Subsequently, 1,802 upregulated and 1,175 downregulated DEGs were found in the early gall stage (compared to E-GL, Fig. 5A). In the L-G, 1,211 upregulated DEGs and 653 downregulated genes were identified (compared to L-GL, Fig. 5A). Among these, 1,159 DEGs were identified at both developmental stages. Early-stage galls had 1,818 DEGs that were distinct from mature gall tissues, with 705 DEGs that were not found in E-G (Fig. 5B). Comparing early and late gall tissues (L-G/E-G), 800 DEGs were highly expressed in the E-G, whereas 444 were upregulated in mature gall tissues (L-G) (Fig. 5A).

### Gene ontology and KEGG pathway enrichment of DEGs

The enrichment of the upregulated and downregulated DEGs in the BP terms is listed in Table 1 (for details, see Datasheet S3). The significant BP terms of the upregulated DEGs that were enriched specifically in the E-G group were related to the movement of the substrate (GO:0007018; GO:0007017; GO:0006928), ROS response (GO:0098869; GO:1990748; GO:0097237; GO:0072593), and cell cycle (GO: 0022402; GO:0007049).

**Table 1:** Enrichment of GO biological process (BP) terms of DEGs in early and late stages of gall tissues (FDR < 0.05)

		Enriched in Early stage galls (E-G)	Enriched in late stage galls (L-G)
**Up-regulated**	**Stage specific**	GO:0007018 Microtubule-based Movement	GO:0009056 Catabolic Process
		GO:0006468 Protein Phosphorylation	GO:0000272 Polysaccharide Catabolic Process
		GO:0007017 Microtubule-Based Process	GO:1,901,575 Organic Substance Catabolic Process
		GO:0006928 Movement of Cell or Subcellular Component	GO:0016052 Carbohydrate Catabolic Process
		GO:0098869 Cellular Oxidant Detoxification	GO:0009057 Macromolecule Catabolic Process
		GO:1,990,748 Cellular Detoxification	GO:0005976 Polysaccharide Metabolic Process
		GO:0097237 Cellular Response to Toxic Substance	GO:0017144 Drug Metabolic Process
		GO:0072593 Reactive Oxygen Species Metabolic Process	GO:0045488 Pectin Metabolic Process
		GO:0022402 Cell Cycle Process	GO:0010393 Galacturonan Metabolic Process
		GO:0007049 Cell Cycle	GO:0016042 Lipid Catabolic Process
		GO:0007059 Chromosome Segregation	GO:0046514 Ceramide Catabolic Process
		GO:0006811 Ion Transport	GO:0043086 Negative Regulation of Catalytic Activity
		GO:0007178 Transmembrane Receptor Protein Serine/Threonine Kinase Signaling Pathway	GO:0055114 Oxidation-Reduction Process
		GO:0007167 Enzyme Linked Receptor Protein Signaling Pathway	
	**Shared**	GO:0016310 Phosphorylation	
		GO:0042737 Drug Catabolic Process	
		GO:0046777 Protein Autophosphorylation	
		GO:0042744 Hydrogen Peroxide Catabolic Process	
		GO:0017001 Antibiotic Catabolic Process	
		GO:0042743 Hydrogen Peroxide Metabolic Process	
		GO:0051187 Cofactor Catabolic Process	
		GO:0005975 Carbohydrate Metabolic Process	
		GO:0045490 Pectin Catabolic Process	
		**Enriched in Early stage galls (E-G)**	**Enriched in late stage galls (L-G)**
**Down-regulated**	**Stage specific**	GO:0048544 Recognition of Pollen	No match
		GO:0008037 Cell Recognition	
		GO:0010207 Photosystem II Assembly	
		GO:0009875 Pollen-Pistil Interaction	
	Shared	GO:0006468 Protein Phosphorylation	
		GO:0016310 Phosphorylation	
		GO:0000413 Protein Peptidyl-Prolyl Isomerization	
		GO:0018208 Peptidyl-Proline Modification	

In contrast, the downregulated DEGs were significantly categorized into BP terms of pollen recognition (GO:0048544) and pollen-pistil interaction (GO:0009875), as well as PSII assembly (GO:0010207). Among the L-G-specific DEGs, upregulated DEGs were enriched in the catabolic processes of various molecules, including polysaccharides, lipids, carbohydrates, and organic substances. The BP term, negative regulation of catalytic activity (GO:0043086), also showed the significance of the upregulated DEGs in the L-G group. This analysis found no enrichment of downregulated DEGs, specifically in the L-G group.

**Fig. 6:**
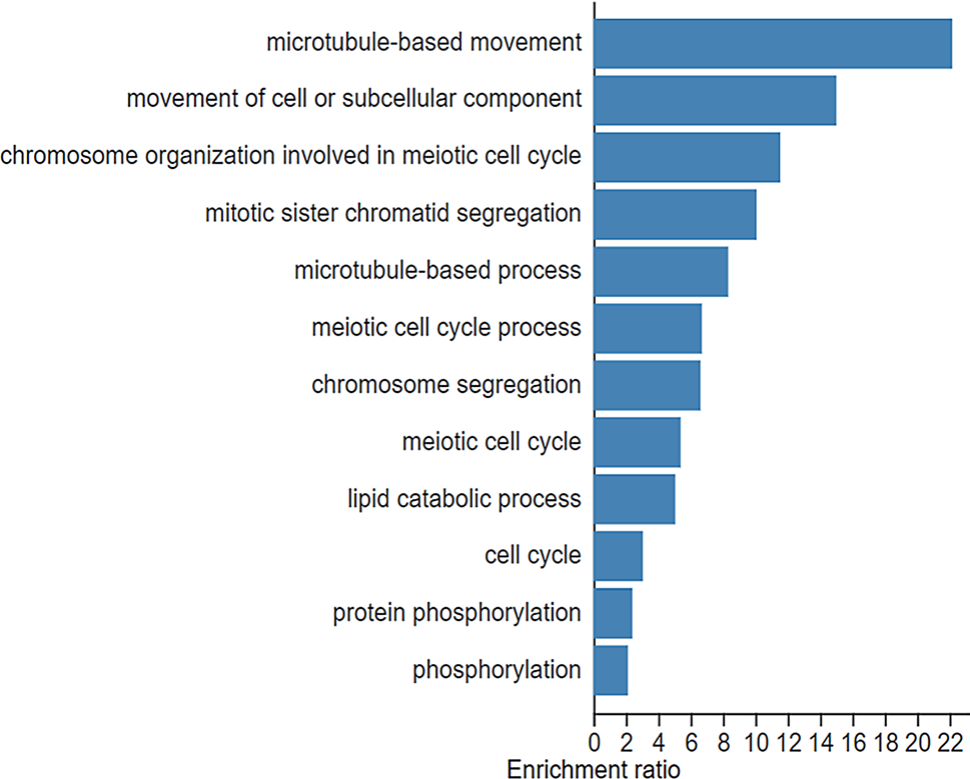
Enrichment of GO Biological process (BP) terms of up-regulated DEGs in early gall tissues (**E**-**G**) when compared to late gall tissue (**L**-**G**)

When subjected to the DEGs from the comparison between two gall stages in GO enrichment analysis, no significant BP term of the upregulated DEGs in L-G was found under the threshold FDR < 0.05. However, upregulated DEGs in E-G were significantly categorized into processes involving the meiotic and mitotic cell cycles (Fig. 6), suggesting a higher cell division activity in the early developmental stages. Additionally, analysis of DEG enrichment in the KEGG pathway also demonstrated that upregulated or downregulated DEGs in the E-G and L-G were involved in metabolizing primary and secondary metabolites (Table 2).

**Table 2:** KEGG pathways enrichment of DEGs in early and late stages of gall tissues (Top 10 ranking)

Pathway categories	Enriched in both E-G and L-G	Gene Set	Enriched specifically in E-G	Gene Set	Enriched specifically in L-G	Gene Set
**Up-regulated**	Phenylpropanoid biosynthesis	*ath00940*	Base excision repair	*ath03410*	Fructose and mannose metabolism	*ath00051*
	Pentose and glucuronate interconversions	*ath00040*	Starch and sucrose metabolism	*ath00500*	Taurine and hypotaurine metabolism	*ath00430*
	Sphingolipid metabolism	*ath00600*	Indole alkaloid biosynthesis	*ath00901*	Tyrosine metabolism	*ath00350*
	Biosynthesis of secondary metabolites	*ath01110*	Lysine degradation	*ath00310*	Other glycan degradation	*ath00511*
					Isoquinoline alkaloid biosynthesis	*ath00950*
					Ribosome	*ath03010*
**Down-regulated**	Glycine, serine and threonine metabolism	*ath00260*	Glycolysis / Gluconeogenesis	*ath00010*	Phenylpropanoid biosynthesis	*ath00940*
	Glyoxylate and dicarboxylate metabolism	*ath00630*	Carbon fixation in photosynthetic organisms	*ath00710*	One carbon pool by folate	*ath00670*
	Biosynthesis of secondary metabolites	*ath01110*	Fatty acid degradation	*ath00071*	Thiamine metabolism	*ath00730*
	Carbon metabolism	*ath01200*	Ubiquitin mediated proteolysis	*ath04120*	Nitrogen metabolism	*ath00910*
	Nicotinate and nicotinamide metabolism	*ath00760*				
	Tyrosine metabolism	*ath00350*				

### Identification of proteins in early gall tissues

By examining the selected proteins with increased abundance in early gall tissues, 23 proteins were identified with at least two sequenced peptides identified in the database (Table 3; for details, see Table S3). Half of these isolated proteins, including HLP1, calmodulin 7, actin 4, and eIF2-beta, were annotated as factors involved in regulating the cell cycle or cellular development. Other screened proteins were annotated as defense or malate/fatty acid metabolic components. A primary auxin response element, FQR1, was also found in early gall stages.

**Table 3:** Identification of the up-regulated proteins in early stage galls (E-G)

Accession	Description	MW [kDa]/pI	Main function
NP_195634.1	NmrA-like negative transcriptional regulator family protein (PCBER1)	34.1/6.8	Defense
NP_001331372.1	RNA-binding (RRM/RBD/RNP motifs) family protein (RBGD5); (HLP1)	40.7/9.3	Cell cycle
NP_200261.1	Flavodoxin-like quinone reductase 1 (FQR1)	21.7/6.3	Auxin response
NP_849940.1	NAD(P)-binding Rossmann-fold superfamily protein (ENR1)(MOD1)	41.1/8.9	Malate & fatty acid
NP_188902.1	20 S proteasome beta subunit D1 (PBD1)	22.5/6.3	Protein catabolism
NP_173786.1	Oxidoreductase, zinc-binding dehydrogenase family protein (AOR)	40.9/8.3	Detoxification
NP_200959.2	GroES-like zinc-binding alcohol dehydrogenase family protein	43.9/8.3	Development
NP_974817.1	Eukaryotic translation initiation factor 2 beta subunit (EIF2 BETA)	30.5/7.1	Development
NP_563780.1	Light-dependent short hypocotyls-like protein (LSH6)	21.5/9.4	Development
NP_001078474.1	Serine/arginine-rich 22 (SRZ-22)	22.4/11.	Cell cycle
NP_171812.1	Photosystem I subunit D-2 (PSAD-2)	22.2/9.7	Photosynthesis
NP_850660.1	Histone superfamily protein	11.4/11.	Development
NP_200679.1	Rotamase CYP 7 (ROC7)	21.9/9.0	Development
NP_182287.1	UDP-xylose synthase 4 (UXS4)	49.9/8.9	Cell wall
NP_564762.2	NAD(P)-linked oxidoreductase superfamily protein	37.8/6.3	Defense
NP_001031860.1	Peroxisomal NAD-malate dehydrogenase 2 (PMDH2)	34.9/7.6	Malate & fatty acid
NP_567536.1	RNA-binding (RRM/RBD/RNP motifs) family protein (IRP3)(BPL1)	33.5/6.1	Defense
NP_193486.1	RAB GTPase homolog 1 C (RAB1C)(RABD2C)	22.3/5.4	Development
NP_175350.1	Actin 8	41.8/5.5	Development
NP_199147.1	Lactate/malate dehydrogenase family protein (c-NAD-MDH2)	35.6/6.7	Malate & fatty acid
NP_001189892.1	Aldolase-type TIM barrel family protein (GOX2)	40.2/8.9	Defense
NP_189967.1	Calmodulin 7	16.8/4.2	Development
NP_001078769.1	Actin 4	41.7/5.5	Development

To examine whether there was any over-representation of the identified proteins, the accessions of identified proteins were converted into Arabidopsis gene ID (AGI), and the BP of GO was performed. The top-ranked BP terms were the regulation of photoperiodism, lignin metabolism, mRNA polyadenylation, and oxidation-reduction processes (Fig. S2A).

**Fig. 7:**
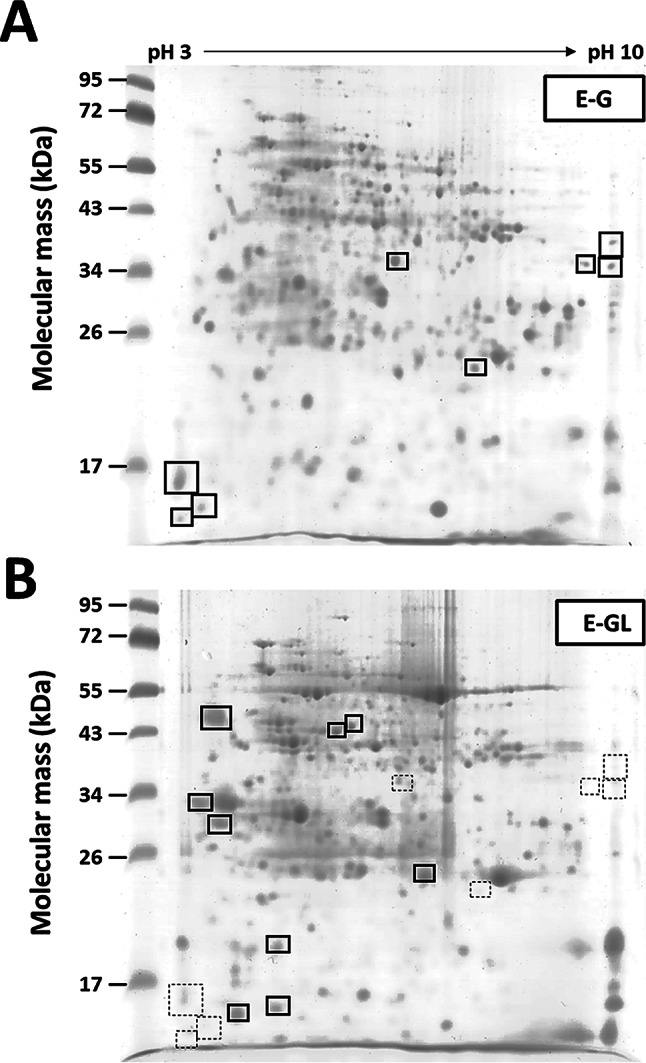
2-D electrophoresis maps comparing proteins isolated from galls and leaves. (**A**) 2-DE gel of total proteins from early-stage gall tissues (**E**-**G**). The indicated regions by boxes are selected for regarding as up-regulated protein molecules in E-G. (**B**) 2-DE gel of total proteins from the galled leaves corresponding to early gall stage. The indicated regions by boxes (solid border) are selected for regarding as down-regulated protein molecules in E-G. Boxes with dotted border indicate the region selected in panel A. Selected protein spots were identified by LC-MS/MS analysis (Table 3, for detail, see Table S3-S4)

2-DE also revealed downregulated proteins in E-G by identifying the protein spots in infected leaves (E-GL) that were absent or decreased in gall tissues (E-G) (Fig. 7B). Approximately 70% (33 of 48) of the identified proteins were related to photosynthesis (chloroplast structure, light reaction, and carbon fixation; Table S4). Two identified proteins were involved in photorespiration (HPR and GDCH). All downregulated proteins identified by 2-DE are listed in Table S4. GO analysis of the downregulated proteins through GO demonstrated top-ranked BP terms, including reductive pentose-phosphate cycle, dark reaction, and carbon fixation (Fig. S2B).

### Contents of photopigments and polyphenols

**Fig. 8:**
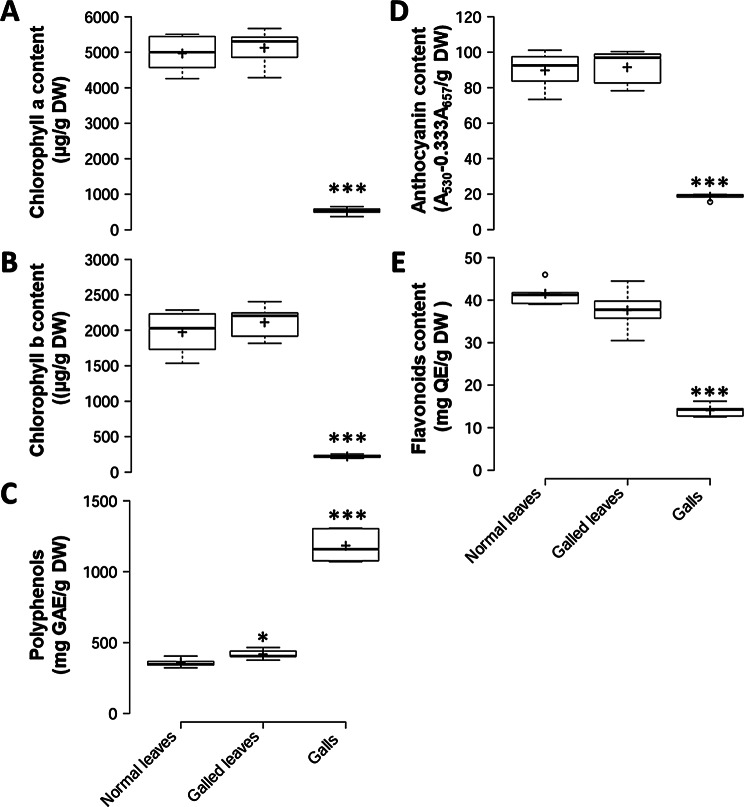
Contents of photopigments and polyphenols in galls and leaves. *Significant difference compared to normal leaves (Student’s *t*-test; *, *p* < 0.05; ***, *p* < 0.001). Sample number are in Chl a and Chl b analysis, *n* = 6; in polyphenol, athocyanin and flavonoid analysis, *n* = 5

Given the demonstrated role of photopigments in modulating plant responses to biotic stress, the contents of chlorophyll a, chlorophyll b, and anthocyanins in gall and leaf tissues were analyzed. Our results showed a significant reduction in the levels of chlorophyll a, chlorophyll b, and anthocyanins in gall tissues (Fig. 8A-B, D). However, no statistical difference in photopigment content was observed between galled leaves and normal uninfected leaves.

Reports suggesting that polyphenols confer higher antioxidant capacity to gall tissues prompted us to analyze the levels of polyphenols and flavonoids in psyllid galls. Compared to normal leaves, galled leaves exhibited higher levels of polyphenols, with a more significant increase observed in gall tissues (Fig. 8C). In contrast, the content of flavonoids was found to be lower in gall tissues (Fig. 8E).

### ROS detection of gall and leaves

**Fig. 9:**
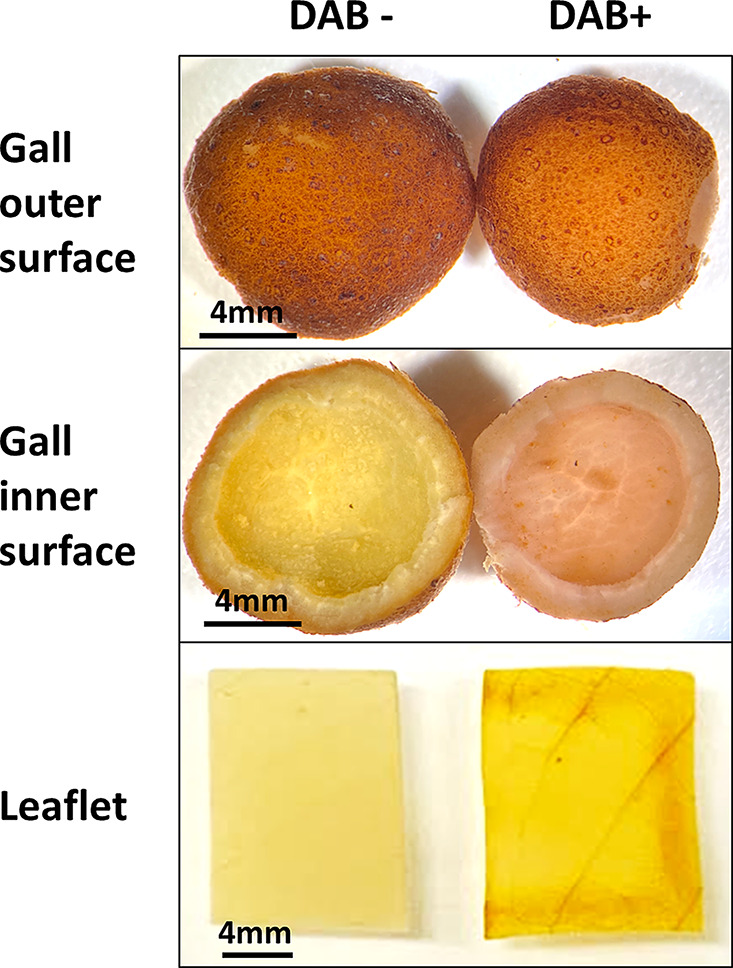
Localization of H_2_O_2_ by DAB-mediated tissues printing in galls and galled leaves. The outer and inner surface of identical DAB- or DAB + galls were showed in upper and middle panels

DAB staining was used to assess the accumulation of hydrogen peroxide, an ROS, in gall tissue. The areas exhibiting accumulation showed a distinct reddish-brown staining pattern. As shown in Fig. 9, staining was observed on the inner surface of the gall tissues, suggesting ROS accumulation within the gall tissues. In contrast, a brown precipitate was observed only at the sites of injury along the leaf-cutting edge. No precipitated signals were found in the galls or leaflets treated with the DAB-free staining solution.

## Discussion

In the present study, we demonstrated the molecular regulation in early- and late-stage psyllid galls, highlighting the expression features of defense-related and signaling-related genes and proteins in developing gall tissues, as well as the nutrient source-oriented shift of gene expression in mature galls. Galls serve as both a habitat and a food source for the insect larvae, providing them with protection from predators and environmental stressors. Within leaf galls, there are increased levels of soluble proteins and other nutrients that support the metabolic needs of the developing insects (Stone and Schönrogge 2003; Oliveira and Isaias 2010). The stress stimuli from continuous larval feeding lead to the alteration of normal plant developmental processes, resulting in the maintenance and growth of the gall structure (Allison and Schultz 2005). The feeding biology of psyllid in gall tissue was described thoroughly in Sharma and Raman (2022), indicating a feeding strategies of larvae on undifferentiated parenchyma made of thin cell walls. This activity thus induces ongoing stress in host plant. Data from this study provided molecular evidence revealing defense response against stress, particularly oxidative stress, in developing gall tissues.

### Defense responses in galls

Defensive regulation plays a considerable role in the plant’s response against galling invasions. Reports have demonstrated the accumulation of secondary metabolites like polyphenols in the infected tissues in order to protect plant cells from damage caused by the oxidative stress associated with herbivore attack. (Taper and Case 1987; Guedes et al. 2019). In this study, the contents of polyphenols were three fold higher in gall tissues, indicating an adaptive plant response to heavily attack with increased defensive needs (Fig. 8C). The protection of non-galled region of leaflet is also needed since the contents of polyphenols in the galled leaves were also raised 17%.

Flavonoids are a type of polyphenols known for pigmentation and antioxidant properties. Our data showed that, in contrast to those of other polyphenols, contents of flavonoids were significantly decreased in gall tissues (Fig. 8E). The contents of anthocyanin, another compound belong to the flavonoid class, were also declined in galls (Fig. 8D). In the study of *Tilia platyphyllos* (Malvaceae), amount of anthocyanin was elevated in the mite galls. Therefore, our result suggests the psyllid galls of *M. kusanoi* might use other phenolic compounds to mitigate the damage after infection. However, it may also suggest that the flavonoids in gall tissues were consumed to synthesize downstream derivatives according to the literatures that have demonstrate the distribution of flavonoid derivatives in gall tissues of *Piptadenia gonoacantha* (Fabaceae) and *Nothofagus obliqua* (Nothofagaceae) (Bedetti et al. 2014; Guedes et al. 2022).

Defensive pathways are linked to the defensive phytohormones jasmonic acid (JA) and salicylic acid (SA), which are often evoked when plants encounter herbivores and pathogens (Zhou et al. 2020). In aphid gall tissue, the expression of genes related to JA and SA-response is upregulated (Hirano et al. 2020). In the psyllid galls examined in the present study, however, no enrichment was assigned to the JA- or SA-related annotations (Fig. 3). Instead, enrichment of phenylpropanoid biosynthesis was found in the upregulated DEGs in both early and mature gall tissues. Phenylpropanoid compounds protect plants against microbial attack by eliciting various defense mechanisms, including callose deposition, cell wall lignification, and the production of antimicrobial substances (Dixon et al. 2002; Yadav et al. 2020). A recent study on lilac (*Syringa pinnatifolia*) showed that the phenylpropanoid pathways accumulate after pathogen infection (Gao et al. 2022). This study also proposes that the increased lignans are biosynthesized mainly through phenylpropanoid pathways during the plant immune response. In our study, the biosynthesis of lignans in the psyllid gall tissues was implied by the presence of PCBER1, a crucial enzyme that activates the biosynthesis of secondary metabolites (Table 3) (Nuoendagula et al. 2015). Further content analysis of the metabolite involved in phenylpropanoid biosynthesis pathway will be able to understand the contribution of flavonoids and the derivatives. Considering the pathways of biosynthesis metabolites were found to be either up-regulated or down-regulated in gall tissues (Table 2), examining the metabolomics differences between leaves and gall tissues will also help to evaluate the strategies in plant defense against psyllid infestation.

### Traits of ROS in galls development

Hypersensitive cell death may be another major phenomenon that occurs in the psyllid gall tissues. Both young, developing, and mature galls exhibited upregulated genes assigned to the sphingolipid metabolic pathway (Table 2). Bioactive sphingolipids have been suggested to be involved in plant programmed cell death (PCD) and are implicated in plant defense and the innate immune system (Berkey et al. 2012). PCD is one of the consequences of elevated ROS (Morel and Dangl 1997). Therefore, we hypothesized that the enrichment of sphingolipid metabolism-related genes in gall tissues might be caused by the increasing levels of ROS, whose metabolic processes were also found to be enriched in DEGs (Table 1, GO:0075293).

Furthermore, the identification of BPL1 (IRP3) in early gall tissues also supports the occurrence of plant hypersensitivity responses (Table 3). A previous study demonstrated that BLP1 is an important binding partner of Arabidopsis accelerated cell death11 (ACD11) and can stabilize the activity of ACD11 (Li et al. 2019). ACD11 is a sphingosine transfer protein whose absence induces PCD and defense responses in Arabidopsis (Brodersen et al. 2002). The synthesis of BPL1 might facilitate the metabolic pathway of sphingolipid metabolism and regulate ROS homeostasis.

Speculating that ROS was the key factor that initiated and regulated gall development in our study might be tempting. In this study, the presence of ROS accumulation within the gall tissues is shown in Fig. 9. Plants generate ROS to regulate defense responses and programmed cell death under unfavorable conditions (Schippers et al. 2016), as in our case, with psyllids ovipositing on leaves. Early responses to ROS signals include fluctuating protein phosphorylation, elevated Ca^2+^ influx, and activation of MAP kinases (MAPKs) (Benschop et al. 2007). Early-stage galls were enriched in phosphorylation- or ion-transport-related genes (Table 1). DEGs involved in the serine/threonine kinase signaling pathway, to which MAPKs belong, were also enriched in E-G. The increased protein levels of Ca^2+^ carrier CAM7 in E-G may further consolidate the impact of ROS signaling. The habitation of insect larvae in leaves is thought to induce long-term ROS signaling, profoundly changing the phenotype of leaf tissues through dramatic transcriptional reprogramming (Zandalinas et al. 2019). Additionally, a notable increase in salicylic acid (SA) was found in early stage galls but not in mature galls (data not shown). This may reflect the dominance of ROS-related metabolic processes in developing gall tissues.

Developmental phytohormones, such as auxins and cytokinins, interact with ROS to regulate plant morphogenesis (Xia et al. 2015; Zwack et al. 2016). The upregulation of auxin- and cytokinin-responsive genes, as well as the accumulation of IAA and CKs in gall tissues, are induced by midges, cynipids, sawflies, and psyllids (Yamaguchi et al. 2012; Bailey et al. 2015; Hearn et al. 2019; Shi et al. 2019). These results suggest that phytohormones are key regulators of insect galling for the manipulation of plant tissues and organs (Dodueva et al. 2020). In our examination of psyllid galls, although the concentrations of auxins and CKs increased in gall tissues (Fig. S3), no DEG enrichment was assigned to functional categories related to phytohormones and their responsive elements. Therefore, ROS may be an upstream factor that crosstalk with phytohormones to promote the growth of abnormal gall tissues (Tognetti et al. 2017).

### Gall development (reproductive and phytohormones)

Based on our results, genes and proteins associated with flowering or seedling development may contribute significantly to the development of gall tissues on leaves. Over-representation analyses of all annotated genes and DEGs showed that early gall tissues were enriched with genes that regulate the cell cycle and organization, consequently increasing the expression of signaling-associated genes (Fig. 3; Table 1; Fig. 4). These functions suggest that developing gall cells undergo several processes linked to mitosis, meiosis, and microtubule-based movement (Fig. 6). Our exploration of elevated proteins in E-G suggested that the RNA-binding protein HLP1 and the serine/arginine-rich (SR) protein SRZ-22 may participate in these processes (cell cycle) (Table 3).

In the 2-DE protein examination, half of the identified proteins governed the development of different plant parts, especially reproductive tissues (Table 3). In the early gall stage, the amount of actin 4, which is predominantly expressed in developing and reproductive tissues, such as ovules and pollen, was elevated (Holmes-Davis et al. 2005). The Rab GTPase RAB1C (RABD2C, (Peng et al. 2011), which regulates pollen tube growth, was also expressed in developing gall tissues. The earlier mentioned SRZ-22 interacts with cyclin-dependent kinase G1 (CDKG1) as a complex that regulates pollen wall formation (Huang et al. 2013). Our transcript and protein data suggest that psyllid infestation and parasitism triggered the reproductive status of leaves and influenced cell proliferation to form galls.

Recent transcriptomic studies of insect galls have revealed the developmental machinery of different gall types. A survey of aphid galls on Chinese sumac showed that genes involved in floral organ development were significantly enriched in gall tissues (Hirano et al. 2020). Another transcriptomic analysis of aphid galls on grapevine leaves demonstrated that gene expression patterns in the gall tissues were manipulated to form carpels (Schultz et al. 2019). Alternatively, our examination implied a psyllid-induced redirection of leaf structure toward the pollen structure. Notably, the DEGs assigned to the GO BP terms pollen recognition and pollen–pistil interactions were downregulated in early gall tissues (Table 1). As there was no actual production or adhesion of pollen, the repression of pollen recognition and interaction might suggest the prevention of further pollination, and other reproductive processes were induced either by the host plant (protection) or by gall inducers (manipulation).

In this study, a scheme in which plants regulate seedling development was proposed as another gall-inducing/developing mechanism. Calmodulin 7 (CAM7), LIGHT-SENSITIVE HYPOCOTYLS 6 (LSH6), and eIF2β were suggested to be involved in seed development and were highly expressed in early gall tissues (Table 3). CAM7 is a versatile calcium-binding protein that acts as an upstream regulator of various cellular activities via Ca^2+^ signaling (Perochon et al. 2011). In the present study, although no significant differences were observed in the Ca^2+^ concentrations between galls and galled leaves, notably, the Ca^2+^ content was generally higher in galls than in galled leaves (Fig. 8). The regulation of Ca^2+^ within plant cells is involved in many physiological functions, making it a highly intricate process. However, these findings warrant further investigation. In addition, CAM7 acts as a transcription factor in concert with HYPOCOTYL5 (HY5) bZIP protein to promote photomorphogenesis via a light signaling pathway during seedling development (Abbas et al. 2014). LSH6 was presumably assigned to respond to light stimuli by its paralogous protein LSH1, which is coupled with a phytochrome to mediate light-dependent seedlings (Zhao et al. 2004). CAM7 and LSH6 in early gall tissues suggest that an upstream stimulus initiates photomorphogenic pathways during gall development. Upstream signals are likely stress-related to the immune-responsive characteristics of CAM7 (Tian et al. 2019; Basu et al. 2021).

Phytohormones are key factors that regulate gall development. Gall-inducing insects contain phytohormones, such as auxins and cytokinins that manipulate gall formation (Yamaguchi et al. 2012; Tanaka et al. 2014; Hirano et al. 2020). Transcriptome analysis of phytohormone-related genes in cynipid and aphid galls has revealed significant upregulation of auxin response pathways (Hearn et al. 2019; Hirano et al. 2020). Gall tissues induced by psyllids on Myrtaceae leaves also show increased expression of auxin response elements (Bailey et al. 2015). In our study, no enrichment of DEGs was assigned to a particular GO BP term related to auxin or other phytohormones (Table 1). This could be caused by different parameter settings in our study and others or by different regulatory networks exhibited in different host plants. In this study, we identified an auxin response factor, FQR1, whose expression increased in early gall tissues (Table 3). However, this primary auxin-response gene might not directly participate in gall development but may be involved in the detoxification process against pathogens and gall inducers (Laskowski et al. 2002; Heyno et al. 2013). DEG enrichment of the ROS detoxification function in the early gall stage supports the role of FQR1 in gall tissue (Table 1).

### Source-sink allocation

Photosynthetic deficiency is a common characteristic of insect leaf galls. The activities of PSII and the content of photopigments were significantly reduced in the gall tissues, as shown in previous studies (Huang et al. 2014a, b). Transcriptomic analysis has also demonstrated the downregulation of photosynthesis-related genes in the leaf gall tissues of *Vitis*, *Litsea* and *Quercus* plants (Nabity et al. 2013; Shih et al. 2018; Hirano et al. 2020; Martinson et al. 2021). In this study, transcriptional alterations during gall formation in two developmental stages were analyzed, and the results showed consolidation of the photosynthesis-related transcriptome profile and photosynthetic physiological traits (Fig. 1). Regarding the enrichment analysis results, the gene sets related to photosynthesis were significantly reduced in gall tissues (Fig. 3). Our 2-DE analyses also demonstrated that many reduced or absent proteins were associated with light reactions, carbon fixation, and chloroplasts (Table S3).

## Conclusions

In conclusion, we used transcriptomic analyses to examine gene enrichment in gall tissues and used a proteomic approach to identify molecules that might participate in the gall-forming process. Here, we presented a case in which ROS-related stress response was predominant during early gall development. Further studies should analyze the effects of ROS inhibition or overload by through the treatment of ROS scanvenger (like ascrobic acid) or enhencer (like methyl jasmonate) on the development of gall tissues. Examination of metabolites, especially the biotic defense-associtated molecules like JA and SA, in the developing galls could also provide insights of the regulatory network of gall morphogenesis induced by psyllid.

## Electronic supplementary material

Below is the link to the electronic supplementary material.

Supplementary Material 1

Supplementary Material 2

Supplementary Material 3

Supplementary Material 4

Supplementary Material 5
