# Supplementary material for: Transcriptome and 2-DE proteome analyses reveal defense-associated development in the leaf galls induced by psyllids on Machilus japonica var. kusanoi
Source: Bot Stud. 2025 Jul 14;66:19. doi: 10.1186/s40529-025-00470-2 (PMC12260147; doi:10.1186/s40529-025-00470-2)
Supplement: Supplementary file 4 — Supplementary Material 4 [file 40529_2025_470_MOESM4_ESM.docx]

| **Table S1. Read number in each sample.** | | |  |
| --- | --- | --- | --- |
| **Sample_Name** | **# Reads** | **Avg Read Length** | **% GC** |
| **E-G1** | 4,42,52,204 | 143.09 | 46.42% |
| **E-G2** | 5,37,24,454 | 143.88 | 47.16% |
| **E-G3** | 5,02,17,528 | 144.19 | 46.42% |
| **E-GL1** | 8,47,15,098 | 141.49 | 46.18% |
| **E-GL2** | 7,34,99,444 | 141.64 | 45.83% |
| **E-GL3** | 4,20,01,808 | 140.03 | 46.17% |
| **E-L1** | 6,91,78,088 | 140.96 | 45.69% |
| **E-L2** | 5,17,45,842 | 141 | 45.90% |
| **E-L3** | 3,99,10,968 | 140.79 | 46.21% |
| **L-G2** | 4,31,12,782 | 142.25 | 47.56% |
| **L-G3** | 3,96,04,590 | 142.58 | 47.20% |
| **L-G5** | 4,81,86,078 | 139.45 | 46.86% |
| **L-GL1** | 4,85,28,042 | 140.46 | 45.53% |
| **L-GL2** | 4,56,89,438 | 140.92 | 45.95% |
| **L-GL3** | 5,32,38,234 | 140.58 | 45.77% |
| **L-L1** | 3,77,84,136 | 140.32 | 45.74% |
| **L-L2** | 5,47,10,304 | 141.81 | 46.13% |
| **L-L3** | 8,35,53,246 | 141.86 | 46.11% |
| **Total PE reads** | 96,36,52,284 |  |  |
