# Supplementary material for: Transcriptome and 2-DE proteome analyses reveal defense-associated development in the leaf galls induced by psyllids on Machilus japonica var. kusanoi
Source: Bot Stud. 2025 Jul 14;66:19. doi: 10.1186/s40529-025-00470-2 (PMC12260147; doi:10.1186/s40529-025-00470-2)
Supplement: Supplementary file 5 — Supplementary Material 5 [file 40529_2025_470_MOESM5_ESM.docx]

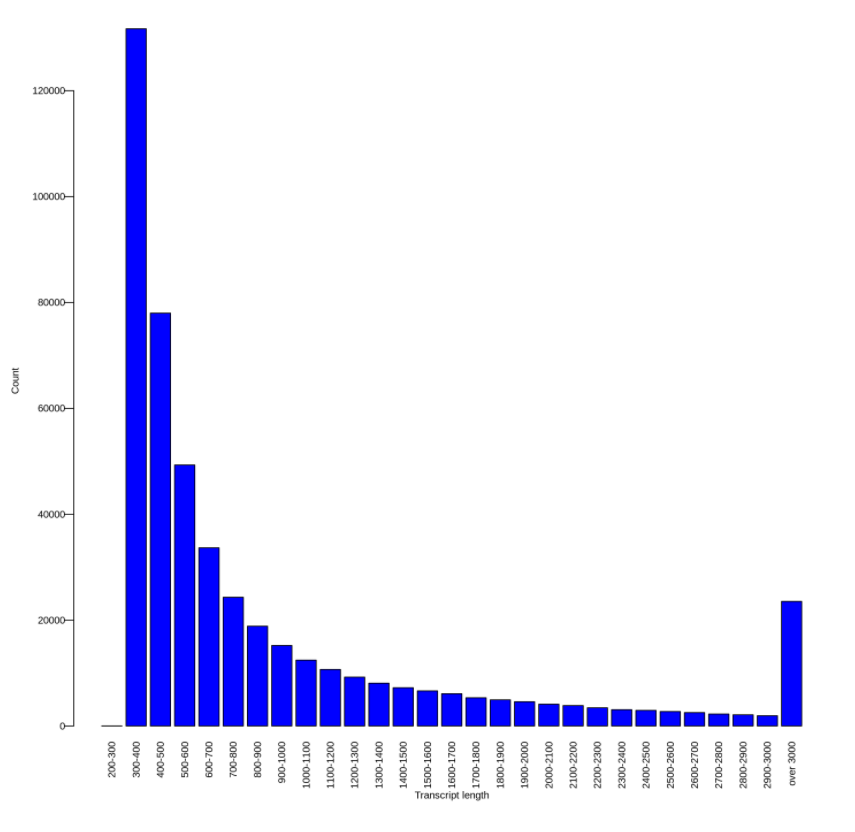
**Supplementary materials**

**Fig. S1.** Distribution of transcript length.

**Fig. S2.** Enrichment of GO BP terms from the gene locus of (A) up-regulated and (B) down-regulated proteins in early stage galls. Enrichment categories was shown in top 10 ranking. Gene locus are listed in supplementary file 2. Navy blue indicates an FDR < 0.05 in the GO terms.


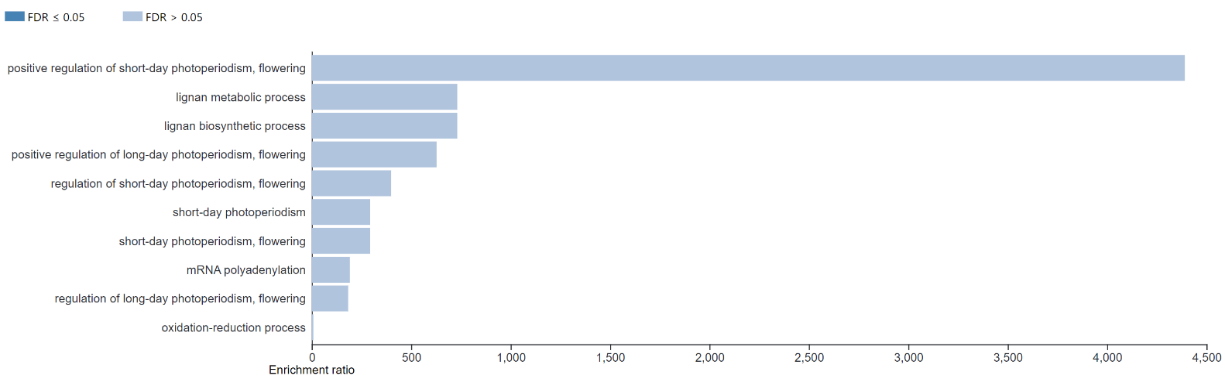

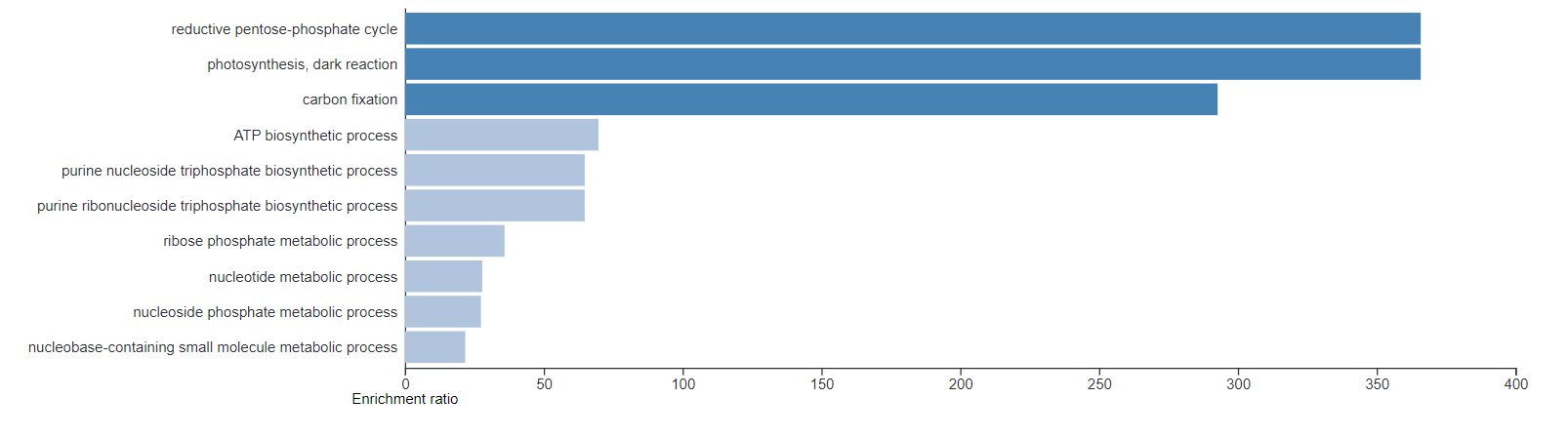


**A**

**B**


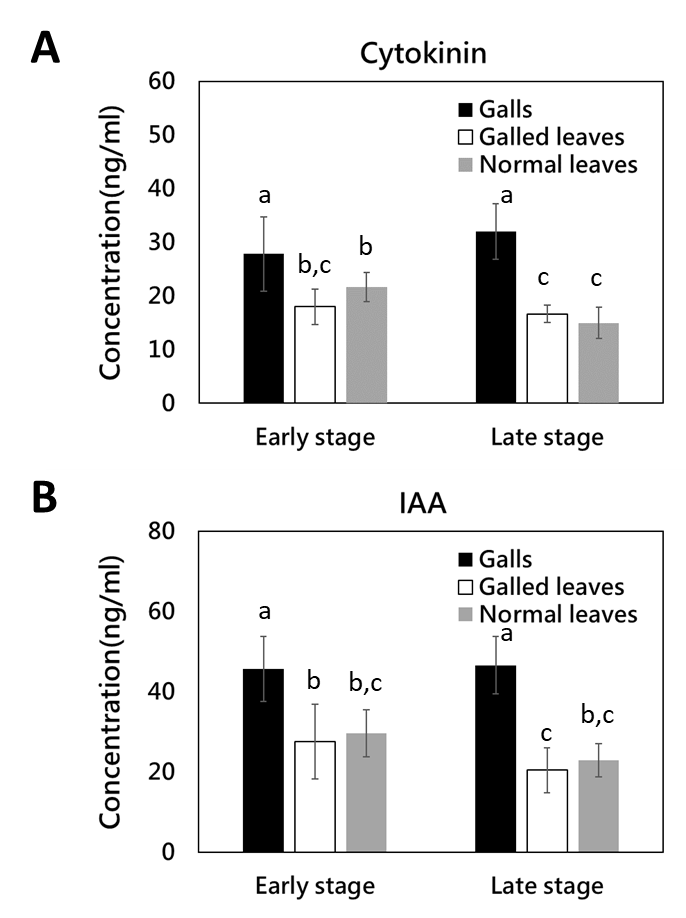


**Fig. S3.** Contents of phytohormones. Contenet of cytokinin (A) and IAA (B) in galls, galled leaves, and normal leaves were analyzed. ^a,b, c^, different group in LSD test (<0.05). Data are presented as mean±sd (n=6). Quantitative detaction were perfromed by ELISA and followed the manufacturer’s protocol (Cytokinin ELISA Kit: Cat No. MBS269996; IAA ELISA Kit: Cat No. MBS269958).
